# Supplementary material for: The antidepressant drug vilazodone is an allosteric inhibitor of the serotonin transporter
Source: Nat Commun. 2021 Aug 20;12:5063. doi: 10.1038/s41467-021-25363-3 (PMC8379219; doi:10.1038/s41467-021-25363-3)
Supplement: Supplementary file 1 — Supplementary Information [file 41467_2021_25363_MOESM1_ESM.pdf]

SUPPLEMENTARY FIGURES AND TABLES for

The antidepressant drug vilazodone is an allosteric inhibitor of the serotonin transporter

Per Plenge[1], Dongxue Yang[2]*, Kristine Salomon[1], Louise Laursen[1], Iris E. Kalenderoglou[1], Amy H. Newman[3], Eric Gouaux[2,4], Jonathan Coleman[2,5,]*, Claus J. Loland[1,]*

[1]Laboratory for Membrane Protein Dynamics. Department of Neuroscience, Faculty of Health and Medical Sciences, University of Copenhagen, Copenhagen, Denmark.

[2]Vollum Institute, Oregon Health & Science University, Portland, Oregon, USA

[3]Medicinal Chemistry Section, Molecular Targets and Medications Discovery Branch, National Institute on Drug Abuse - Intramural Research Program, National Institutes of Health. Baltimore, Maryland, USA

[4] Howard Hughes Medical Institute, Oregon Health & Science University, Portland, Oregon, USA

[5]Department of Structural Biology, University of Pittsburgh, Pittsburgh, Pensylvania, USA.(Present address).

These authors contributed equally: Per Plenge, Dongxue Yang

*Correspondence:

Claus J. Loland, Department of Neuroscience, University of Copenhagen, Denmark, cllo@sund.ku.dk

For technical matters on cryo-EM portion of the work: Jonathan A. Coleman, Department of Structural Biology, University of Pittsburgh, USA, coleman1@pitt.edu

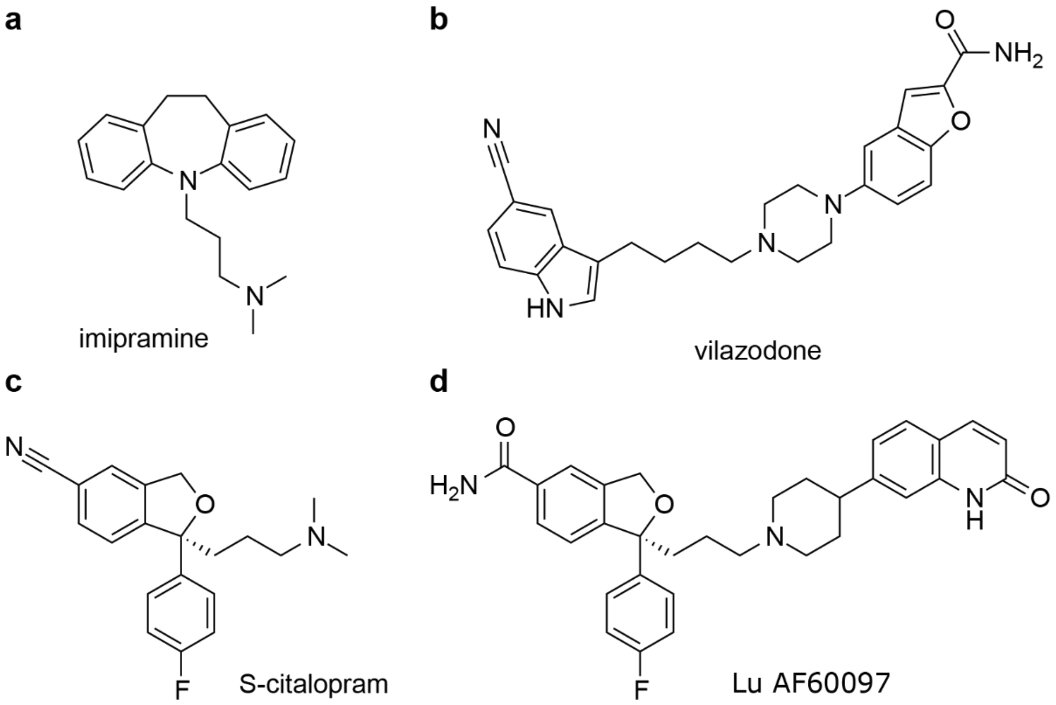

Supplementary Figure 1. Chemical structures of principal ligands. a, Imipramine, IMI (3-(10,11-dihydro-5H-dibenzo[b,f]azepin-5-yl)-*N,N*-dimethylpropan-1-amine). **b,** Vilazodone, VLZ (5-(4-(4-(5-Cyano-1H-indol-3-yl)butyl)piperazin-1-yl)benzofuran-2-carboxamide). **c,** S-citalopram, S-CIT (S-(+)-1-[3-(Dimethylamino)propyl]-1-(4-fluorophenyl)-1,3-dihydro-5-isobenzofurancarbonitrile). **d,** Lu AF60097 ((*S*)-1-(4-Fluorophenyl)-1-(3-(4-(2-oxo-1,2-dihydroquinolin-7-yl)piperidin-1-yl)propyl)-1,3-dihydroisobenzofuran-5-carboxamide).

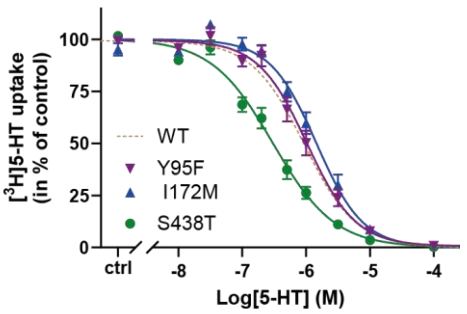

Supplementary Figure 2. 5-HT uptake affinity for S1 mutants. Transport activity for the SERT mutants Y95F (purple inverted triangles), I172M (blue triangles) and S438T (green circles) relative to WT (dotted line, data from Fig 1a). Y95F and I172M have a 5-HT affinity comparable to WT while S438T has a marked increase in affinity. Experiments are performed in triplicates on intact COS7 cells transiently expressing the indicated mutants. Data are means ± S.E. (error bars) of n = 4-5, see Supplementary Table 1 for all quantitative data as well as n-value for each specific experiment. Source data are provided as a Source Data file.

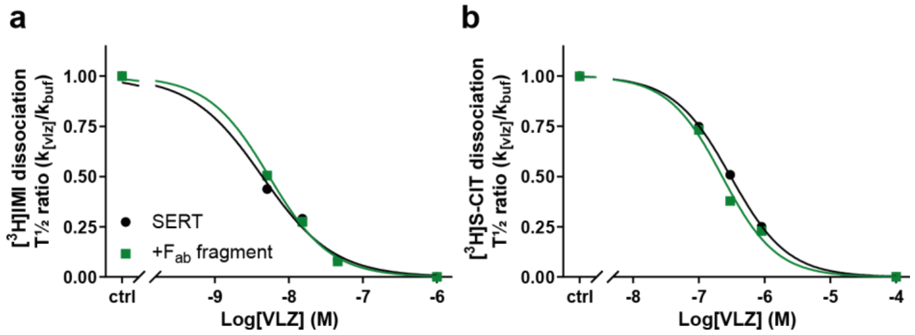

Supplementary Figure 3. Effect of Fab fragment on VLZ binding to SERT. There is no apparent effect on the allosteric potency of VLZ by the addition of Fab fragment. Inhibition of **a,** [^{3}H]IMI or **b,** [^{3}H]S-CIT dissociation from SERT by VLZ, in the absence (black) or presence (green) of 10 μM Fab fragment (chosen to obtain a SERT:Fab concentration of 1:1) targeting the extracellular side of SERT. Experiments are performed on membranes from COS7 cells transiently expressing SERT. Due to the large quantity of Fab fragment required, each experiment was performed only once. Source data are provided as a Source Data file.

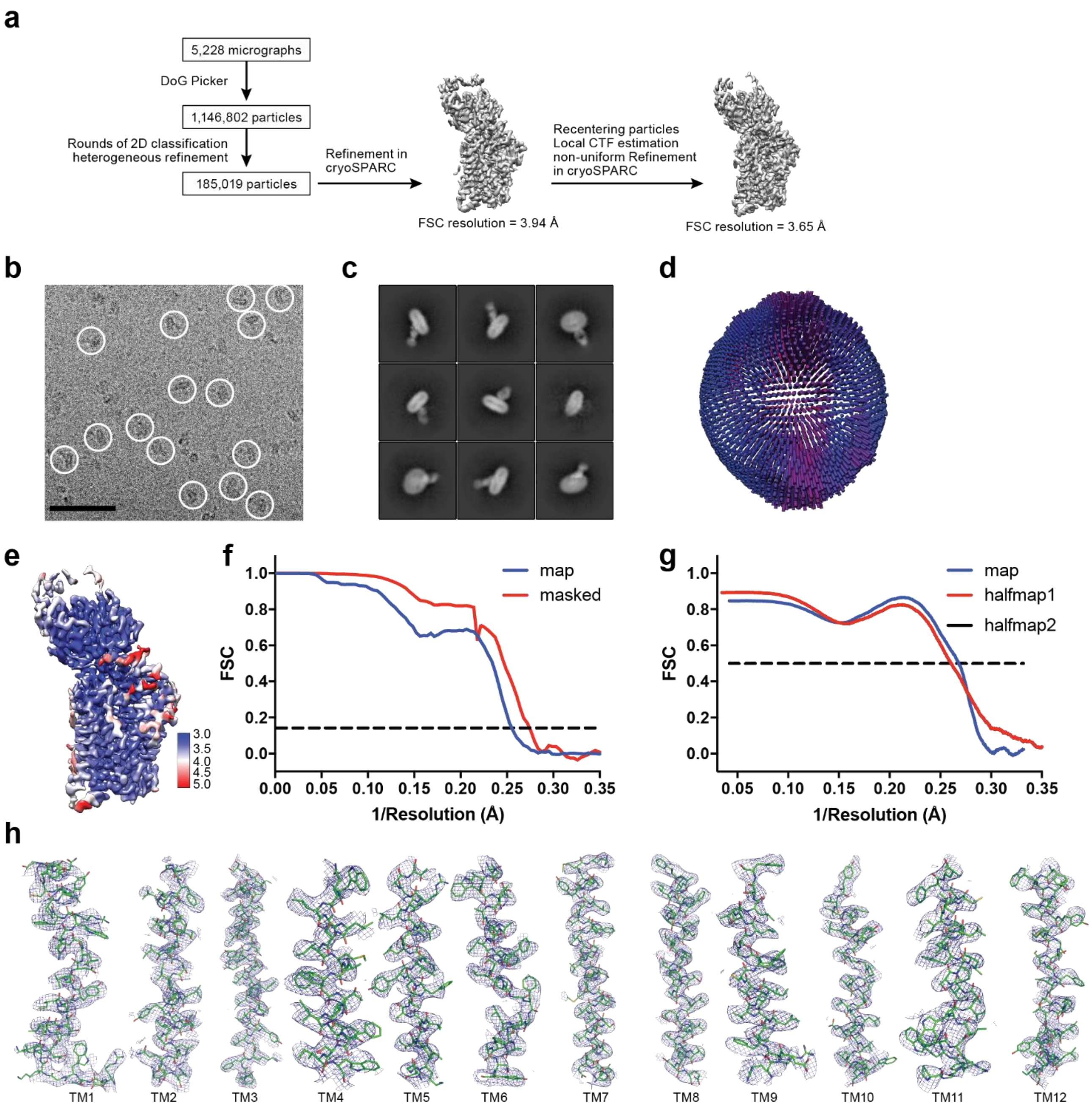
a
5,228 micrographs
DoG Picker
1,146,802 particles
Rounds of 2D classification
heterogeneous refinement
185,019 particles
Refinement in
cryoSPARC
FSC resolution = 3.94 Å
Recentering particles
Local CTF estimation
non-uniform Refinement
in cryoSPARC
FSC resolution = 3.65 Å
b
c
d
e
3.0
3.5
4.0
4.5
5.0
f
map
masked
FSC
1/Resolution (Å)
g
map
halfmap1
halfmap2
FSC
1/Resolution (Å)
h
TM1
TM2
TM3
TM4
TM5
TM6
TM7
TM8
TM9
TM10
TM11
TM12

Supplementary Figure 4. Cryo-EM reconstruction of ΔN72,C13 SERT:15B8 Fab:IMI:VLZ complex. **a,** Work-flow of cryo-EM data processing of the ΔN72,C13 SERT/15B8 Fab complex with IMI/VLZ in the outward-open conformation. After particle picking, particles were sorted using 2D classification. Particles were refined further in cryoSPARC and in *cis*TEM using local refinement, which improved the resolution upon masking of the Fab constant domain and micelle (mask is shown overlaid in yellow on top of the reconstruction). The final reconstructed volume was sharpened using *cis*TEM. **b,** Representative cryo-EM micrograph. Individual single particles are circled in white. Bar equals 50 nm. **c,** 2D class averages after three rounds of classification. **d,** The angular distribution of particles used in the final reconstruction. **e,** Cryo-EM density map colored by local resolution estimation. **f,** FSC curves for cross-validation, the final map (blue), masked SERT-Fab complex (red), and a mask which isolated SERT (black). The low-resolution limit cutoff for refinement was 8.0 Å. **g,** model vs. half map 1 (working, red), half map 2 (free, black), model vs. final map (blue). **h,** Cryo-EM density segments of TM1 - TM12.

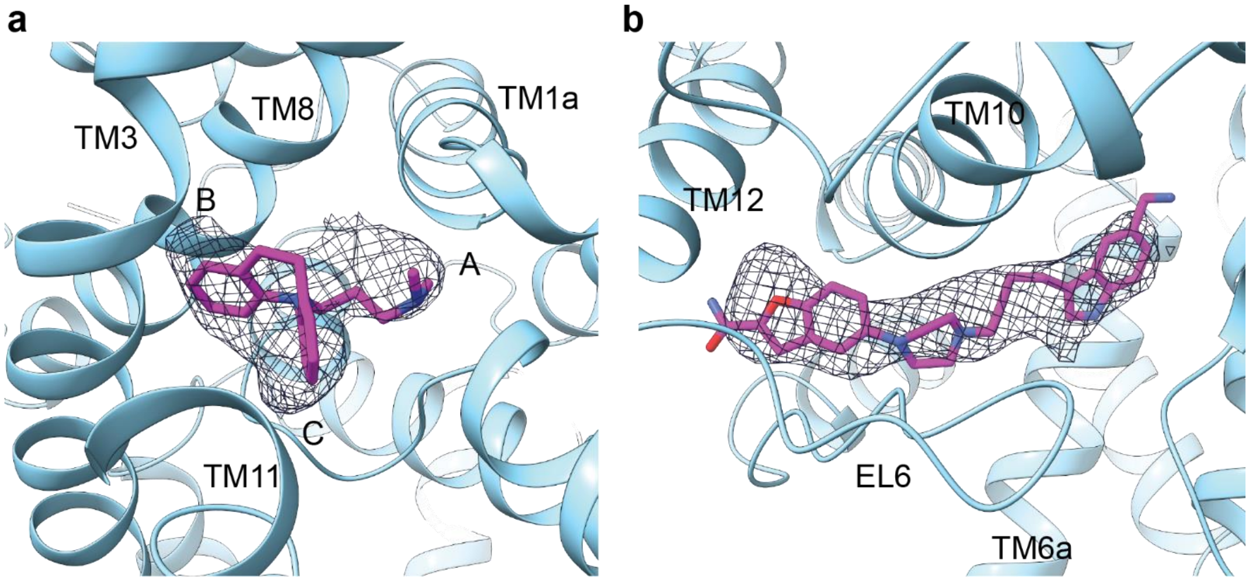

Supplementary Figure 5. Ligand fit with 180° rotation in the central and allosteric sites. a, IMI (violet) is shown fit into a density feature (mesh) at the S1 site with 180° flip and the involved SERT backbone structures (light blue) annotated. **b**, VLZ (violet) is shown fit into a density feature at the allosteric site with 180° rotation with the central SERT backbone structures annotated.

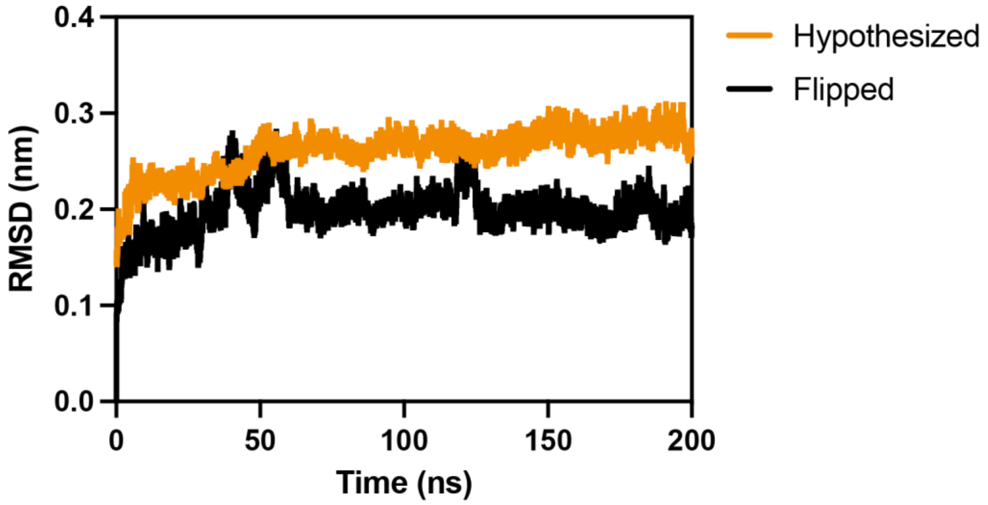

Supplementary Figure 6. Root Mean Square Deviation (RMSD) of the Cα backbone atoms with reference to the initial cryo-EM refined structure. Orange plot corresponds to the system with the hypothesized VLZ pose and black plot corresponds to the system with the flipped vilazodone pose.

Supplementary Table 1. Transport and binding kinetics for SERT WT and S1 site mutants

	5-HT V_{MAX} (pmol/min/10^5 cells)	5-HT K_M (nM)	n	VLZ K_i (nM)	n	S-CIT K_i (nM)	n
WT	26270 ± 1967	873 [781;976]	17	1.06 [0.90;1.25]	11	5.21 [4.19;6.48]	6
Y95F	39484 ± 4081	994 [787; 1260]	4	1.52 [1.27; 1.81]	4	36.7 [31.5; 42.7]	5
I172M	48875 ± 3013	1420 [1150; 1740]	5	2.13 [1.78; 2.56]	4	1000 [798; 1250]	5
S438T	5959 ± 448	291 [237; 357]	4	2.02 [1.63; 2.50]	4	3260 [2150; 4960]	4

5-HT transport is performed on intact COS7 cells transiently expressing SERT WT or mutants. V_{MAX}, K_M and K_i for the indicated compounds are calculated based on IC_{50} and catalytic activity as described in Methods section. Cells are incubated with [^{3}H]5-HT for three minutes. VLZ and S-CIT are added 30 min prior to [^{3}H]5-HT to obtain equilibrium. Data are shown as mean and either ± S.E. (for V_{MAX}) or [S.E. interval], the latter calculated from pIC_{50} ± S.E. All experiments are performed in triplicates. Note that 5-HT data for SERT WT (in gray) are taken from Table 1 shown here for comparison.

Supplementary Table 2. Effect of VLZ on 5-HT transport kinetics

VLZ conc. (nM)	5-HT V_{MAX} (in % of no VLZ)	5-HT K_M (nM)	n
0	95 ± 10	843 ± 214	8
1	93 ± 13	691 ± 216	3
1.9	98 ± 10	1150 ± 277	7
3.0	66 ± 10	824 ± 295	4
3.8	72 ± 17	1220 ± 667	6
6.0	21 ± 6	693 ± 520	3
7.5	18 ± 8	749 ± 817	6
10	11 ± 5	810 ± 779	6
15	3.4 ± 1.2	1270 ± 960	6

Data are calculated from non-linear regression analysis from saturation uptake experiments for [^{3}H]5-HT transport as a function of increasing VLZ concentrations (see Fig. 2a). Experiments are performed on intact COS7 cells transiently expressing SERT WT. Data are performed in triplicates and shown as means ± S.E. V_{MAX} of [^{3}H]5-HT uptake in the absence of VLZ is 4899 ± 312 cpm/min/10^5 cells. Non-specific uptake is determined in the presence of 1 μM paroxetine.

Supplementary Table 3. Cryo-EM data collection, refinement and validation statistics[a]

	#1 (EMDB-23545) (PDB 7LWD)
**Data collection and processing**	
Magnification	77,160
Voltage (kV)	300
Electron exposure (e–/Å^2)	43
Defocus range (μm)	-0.6 to -2.2
Pixel size (Å)	0.648
Symmetry imposed	C1
Initial particle images (no.)	1,146,802
Final particle images (no.)	185,019
Map resolution (Å)	3.65
FSC threshold	0.143
Map resolution range (Å)[b]	5.0-2.9
**Refinement**	
Initial model used (PDB code)	6DZY
Initial model CC	0.73
Model resolution (Å)[c]	3.7
FSC threshold	0.5
Model resolution range (Å)	23.8-3.7
Map sharpening *B* factor (Å^2)	-147.6
Model composition	
Non-hydrogen atoms	6130
Protein residues	769
Ligands (atoms)	68
B factors (Å^2)	
Protein	34
Ligand	34
R.m.s. deviations	
Bond lengths (Å)	0.003
Bond angles (°)	0.577
Validation	
Refined model CC	0.81
MolProbity score	2.03
Clashscore	12.42
Poor rotamers (%)	0.47
Ramachandran plot	
Favored (%)	93.45
Allowed (%)	6.55
Disallowed (%)	0

[a]Data set #1 is the SERT:15B8-Fab:IMI:VLZ complex

[b]Local resolution range.

[c]Resolution at which FSC between map and model is 0.5.
