# Supplementary material for: The antidepressant drug vilazodone is an allosteric inhibitor of the serotonin transporter
Source: Nat Commun. 2021 Aug 20;12:5063. doi: 10.1038/s41467-021-25363-3 (PMC8379219; doi:10.1038/s41467-021-25363-3)
Supplement: Supplementary file 3 — Description of Additional Supplementary Files [file 41467_2021_25363_MOESM3_ESM.pdf]

Description of Additional Supplementary Files

Supplementary Movie 1. Video of the cryo-EM SERT-IMI-VLZ complex MD simulation, with VLZ bound at the hypothesized pose. The refined cryo-EM SERT structure (orange ribbons) was simulated with VLZ (red) at the S2 site and IMI (cyan) at the S1 site, in a POPC:Chol 3:1 lipid bilayer for 200 ns. Video was generated using the VMD Movie Plugin.

Supplementary Movie 2. Video of the cryo-EM SERT-IMI-VLZ complex MD simulation, with VLZ bound at the flipped pose. The refined cryo-EM SERT structure (grey ribbons) was simulated with VLZ (red) at the S2 site and IMI (cyan) at the S1 site, in a POPC:Chol 3:1 lipid bilayer for 200 ns. Video was generated using the VMD Movie Plugin.
