# Supplementary material for: The antidepressant drug vilazodone is an allosteric inhibitor of the serotonin transporter
Source: Nat Commun. 2021 Aug 20;12:5063. doi: 10.1038/s41467-021-25363-3 (PMC8379219; doi:10.1038/s41467-021-25363-3)
Supplement: Supplementary file 6 — Reporting summary [file 41467_2021_25363_MOESM6_ESM.pdf]

nature portfolio

Corresponding author(s): Jonathan A. Coleman
Claus J. Loland

Last updated by author(s): Jun 24, 2021

Reporting Summary

Nature Portfolio wishes to improve the reproducibility of the work that we publish. This form provides structure for consistency and transparency in reporting. For further information on Nature Portfolio policies, see our Editorial Policies and the Editorial Policy Checklist.

Statistics

For all statistical analyses, confirm that the following items are present in the figure legend, table legend, main text, or Methods section.

n/a	Confirmed	
☐	☒	The exact sample size (n) for each experimental group/condition, given as a discrete number and unit of measurement
☐	☒	A statement on whether measurements were taken from distinct samples or whether the same sample was measured repeatedly
☐	☒	The statistical test(s) used AND whether they are one- or two-sided<br>*Only common tests should be described solely by name; describe more complex techniques in the Methods section.*
☒	☐	A description of all covariates tested
☐	☒	A description of any assumptions or corrections, such as tests of normality and adjustment for multiple comparisons
☐	☒	A full description of the statistical parameters including central tendency (e.g. means) or other basic estimates (e.g. regression coefficient) AND variation (e.g. standard deviation) or associated estimates of uncertainty (e.g. confidence intervals)
☐	☒	For null hypothesis testing, the test statistic (e.g. F, t, r) with confidence intervals, effect sizes, degrees of freedom and P value noted<br>*Give P values as exact values whenever suitable.*
☒	☐	For Bayesian analysis, information on the choice of priors and Markov chain Monte Carlo settings
☒	☐	For hierarchical and complex designs, identification of the appropriate level for tests and full reporting of outcomes
☒	☐	Estimates of effect sizes (e.g. Cohen's d, Pearson's r), indicating how they were calculated

Our web collection on statistics for biologists contains articles on many of the points above.

Software and code

Policy information about availability of computer code

Data collection	Prism 9.0, Dog-Picker (https://github.com/craigyk/emtools), CryoSPARC (v3.2), Maestro (release 2021-2), Epik (v2021-2), CHARMM (v36), GROMACS (v2020.3)
Data analysis	Prism 9.0, GROMACS-MMPBSA (v2020.3), ChimeraX (v0.9), Coot (v0.8.9.1), PHENIX (v1.15.2-3472), MolProbity (http://molprobity.biochem.duke.edu/)

For manuscripts utilizing custom algorithms or software that are central to the research but not yet described in published literature, software must be made available to editors and reviewers. We strongly encourage code deposition in a community repository (e.g. GitHub). See the Nature Portfolio guidelines for submitting code & software for further information.

Data

Policy information about availability of data

All manuscripts must include a data availability statement. This statement should provide the following information, where applicable:

- Accession codes, unique identifiers, or web links for publicly available datasets
- A description of any restrictions on data availability
- For clinical datasets or third party data, please ensure that the statement adheres to our policy

A reporting summary for this Article is available as a Supplementary Information file. The source data underlying Figs. 1, 2, 6, Table 1, Supplementary Figs. 2 and 3, Supplementary Table 1 and 2 are provided as a Source Data file. The data for the SERT cryo-EM structure are deposited in the Electron Microscopy Data Bank (EMDB) with accession code EMD-23545. An atomic model of SERT complexed with imipramine, vilazodone and 15B8 Fab are deposited in the wwPDB with accession code 7LWD. Any additional data supporting the findings described in this manuscript are available from the corresponding authors upon request.

Field-specific reporting

Please select the one below that is the best fit for your research. If you are not sure, read the appropriate sections before making your selection.

- [x] Life sciences
- [ ] Behavioural & social sciences
- [ ] Ecological, evolutionary & environmental sciences

For a reference copy of the document with all sections, see nature.com/documents/nr-reporting-summary-flat.pdf

Life sciences study design

All studies must disclose on these points even when the disclosure is negative.

Sample size	Sample size were determined based on previous experience with the pharmacological assays used. See e.g. Plenge et al 2020 Nat. Commun. and Plenge et al 2012, J Biol Chem.
Data exclusions	A data replicate were excluded if Prism 9.0 determined it as an outlier. All excluded data points are shown in the source data file as blue values.
Replication	All the experimental findings herein could be replicated. All experiments were replicated at least three times. The only exception is data shown in Supplementary Figure 3, which is n = 1. The n is listed for each experiment, either in the figure legend or in Table 1 and Suppl. Tables 1 and 2
Randomization	The pharmacological experiments performed herein were performed in a random order. For [3H]5-HT uptake and [3H]S-CIT binding experiments, SERT WT construct was always included for comparison. Otherwise, randomization is not applicable to the data in this manuscript.
Blinding	The investigators were not blinded during data collection, because data analysis is automated so no bias can be applied.

Reporting for specific materials, systems and methods

We require information from authors about some types of materials, experimental systems and methods used in many studies. Here, indicate whether each material, system or method listed is relevant to your study. If you are not sure if a list item applies to your research, read the appropriate section before selecting a response.

Materials & experimental systems

n/a	Involved in the study
[ ]	[x] Antibodies
[ ]	[x] Eukaryotic cell lines
[x]	[ ] Palaeontology and archaeology
[x]	[ ] Animals and other organisms
[x]	[ ] Human research participants
[x]	[ ] Clinical data
[x]	[ ] Dual use research of concern

Methods

n/a	Involved in the study
[x]	[ ] ChIP-seq
[x]	[ ] Flow cytometry
[x]	[ ] MRI-based neuroimaging

Antibodies

Antibodies used	Fab15B8. The dilution is not relevant, it is not being used for WB, IF etc. We have specified the molar ratio of the antibody used to make complex in the methods section.
Validation	Fab15B8 antibody used for crystallization was made in house and has been validated previously: https://www.nature.com/nature/journal/v532/n7599/full/nature17629.html. There is no commercial suppler

Eukaryotic cell lines

Policy information about cell lines

Cell line source(s)	COS-7 cells (Sigma-Aldrich)
Authentication	None of the cell lines used were authenticated
Mycoplasma contamination	All cell lines were tested for mycoplasma contamination on a regular basis. All tests were negative.

Commonly misidentified lines
(See ICLAC register)

none
